# Data-driven climate-smart strategies for boosting crop yield and minimizing greenhouse gas emissions by optimizing cropping systems and fertilization practices

**DOI:** 10.1371/journal.pone.0352144

**Published:** 2026-06-23

**Authors:** Wentao Wu

**Affiliations:** School of Information Science and Technology (School of Artificial Intelligence), Beijing Forestry University, Beijing, China; University of Minnesota, UNITED STATES OF AMERICA

## Abstract

The conflict between food production and environmental protection calls for climate-smart agricultural solutions. This study investigated data-driven climate-smart strategies for optimizing cropping systems and nitrogen management to increase crop yield and cut greenhouse gas emissions in China’s Beijing-Tianjin-Hebei (BTH) region, which is grappling with pronounced climatic and environmental challenges. The study evaluated three cropping systems: spring maize monoculture (M), winter wheat followed by summer maize double-cropping (WM), and a triple-cropping system encompassing winter wheat, summer maize, and spring maize (WMM). Additionally, four nitrogen fertilization treatments were assessed to understand their impacts. The crop climate resilient index was created to identify the optimal management practices. Leveraging the Agricultural Production Systems sIMulator (APSIM) model, this study simulated the daily dynamics of crop yields, soil organic carbon (SOC) content, and nitrous oxide (N₂O) emissions over a comprehensive 40-year period spanning from 1981 to 2020. The findings revealed intriguing insights into SOC dynamics and nitrogen fertilizer efficiency. Across all cropping systems, the SOC content augmented with increased nitrogen application, with peak levels reaching 294.9 kg·ha ⁻ ¹ under the highest fertilization treatment. The N₂O emissions displayed an upward trend over time, positively correlated with escalating fertilizer use. Regarding crop yields, higher nitrogen inputs generally correlated with enhanced productivity. Overall, the WM system, when coupled with F2 treatment (90 kg·ha ⁻ ¹ for wheat and 60 kg·ha ⁻ ¹ for maize), emerged as the optimal scenario, achieving the highest climate resilient index value of 0.65. These findings underscore the profound importance of integrating cropping systems with judicious nutrient management in developing agricultural systems that are adaptive, productive, and environmentally sustainable. By adopting such practices, farmers in the BTH region and similar climates can achieve agricultural clean production that is robust enough to withstand climate variability and contribute to global efforts towards food security and ecological preservation. As the climate continues to evolve, the precision and holistic application of these strategies will be crucial in maintaining the vitality and productivity of agricultural landscapes.

## Introduction

In recent decades, climate warming driven by escalating greenhouse gas (GHG) emissions has emerged as a pressing ecological concern, as highlighted by numerous studies [1–3]. GHGs, particularly nitrous oxide (N_2_O), carbon dioxide (CO_2_), and methane (CH_4_), are key players in the greenhouse effect, necessitating urgent emission reductions to safeguard ecosystems, economies, and global well-being. Notably, N_2_O, a long-lasting stratospheric GHG, poses a more significant threat with a global warming potential 273 times greater than CO_2_ over the next century [1,4]. Agricultural practices stand out as the primary anthropogenic source of N_2_O, accounting for over one-third of global emissions [5–6]. In light of the impending challenges of climate change and food security, sustainable agriculture management must prioritize reducing GHG emissions while enhancing crop productivity [7–9]. Encouragingly, innovative agricultural practices have the potential to mitigate their climate warming impact while maintaining high and consistent crop yields [10]. Therefore, it is imperative to gain a deep understanding of these practices and strengthen the climate resilience of agricultural systems. This ensures that they can continue to support productivity while minimizing environmental impacts, thereby fostering the sustainable development of agriculture [11–13].

Sustainable agricultural management faces a pivotal challenge: striking a balance between reducing GHG emissions and ensuring crop yields amidst increasingly unpredictable climatic conditions. To address this, researchers have explored various agronomic practices, revealing their significant roles in achieving this delicate equilibrium both regionally and globally [14–19]. Central to these practices are optimized fertilization, adjusted cropping systems, and conservation tillage. The strategic application of fertilizers is paramount in contemporary agriculture for boosting crop yields. Studies, such as those by Zhang et al. [14], underscore the crucial role of fertilization. However, merely increasing fertilizer use is not a sustainable solution. Overfertilization can result in reduced nitrogen use efficiency, lower yields, heightened soil GHG emissions, and exaggerated agricultural environmental pollution, as noted by Hartmann et al. [15]. Thus, the focus must be on optimization rather than quantity. Simultaneously, the manipulation of cropping systems and densities offers promising avenues. Proper planting configurations can harness soil temperature and moisture more efficiently, leading to enhanced resource productivity and increased crop yields. Dong et al. [16] found that specific planting densities of 18 × 10^4^ plants·ha^-1^ in a four-row setup and 21 × 10^4^ plants·ha^-1^ in a six-row configuration, yielded optimal results for cotton. These adjustments not only maximized crop output but also balanced resource consumption effectively. Conservation tillage systems, specifically strip tillage, further contribute to sustainability. Wang et al. [17] observed that this practice decreases soil temperature, bolsters soil structure and biodiversity, curbs GHG emissions, and maintains high crop yields. These systems, which contrast with conventional tillage, underscore the importance of soil health and structure in sustainable farming. The existing research base has significantly advanced our comprehension of the intricate relationships between the environment, crops, and climate change. Nevertheless, substantial gaps persist in our understanding of the combined impacts of various cropping systems and management strategies. One critical area that requires further exploration is the synergistic effects of integrated cropping systems and nitrogen management on both climate resilience and emission reduction. Such insights are indispensable for devising adaptive strategies that can enhance agricultural sustainability under the pressure of climate change. In conclusion, while progress has been made, ongoing research and innovation are imperative to refine and tailor agronomic practices to ensure they effectively mitigate GHG emissions while maintaining or even enhancing crop yields. This holistic approach is vital for fostering sustainable agricultural management in an increasingly uncertain climatic landscape.

The Beijing-Tianjin-Hebei (BTH) region located in the northern China, holds a pivotal position as a significant grain production hub. It contributes approximately 12% of the nation’s wheat and 8% of its maize output [20], highlighting its critical role in China’s agricultural sector. In addition, the region stands as one of China’s five major urban agglomerations, driving economic vitality in northern China. However, in recent decades, the BTH area has grappled with several environmental challenges. The interconnected impacts of climate change, accelerated urbanization, and intensive farming practices have led to escalating environmental degradation. These changes pose serious threats to regional food security and ecological balance [21–24]. Consequently, the BTH region serves as an essential case study for devising innovative agricultural systems that can adapt to environmental variations while minimizing GHG emissions. These efforts are crucial for safeguarding both the region’s agricultural productivity and its ecological integrity.

The focus of this research lies in bolstering the climate resilience of China’s agricultural systems. To achieve this, we undertook a multi-faceted approach. Firstly, leveraging the multi-source data and APSIM model, we evaluated how SOC, N₂O emissions, soil greenhouse gas emissions (SGHG), and crop yields varied under three cropping systems and four nitrogen management practices. This quantitative analysis spanned several decades, offering a comprehensive understanding of these dynamics. Furthermore, we developed a crop climate resilient evaluation index, which served as a valuable tool to assess the long‑term impacts of different cropping and nitrogen strategies from 1981 to 2020. This index considered both agricultural productivity and environmental sustainability, ensuring a holistic perspective. Finally, we optimally selected measures to achieve high yields while maintaining low SGHG emissions in the BTH region. This study provides fresh insights and practical recommendations to significantly enhance agricultural adaptability and sustainability under climate change.

## Materials and methods

### Study area

The BTH region, encompassing Beijing, Tianjin, and Hebei Province in China, spans across latitudes 36°42′ to 40°08′ N and longitudes 114°54′ to 117°46′ E. This area experiences a temperate monsoon climate, characterized by an annual temperature range of 11.5°C to 12.5°C, with a cumulative temperature of 10°C reaching 4100°C to 5300°C [25]. Precipitation varies significantly across the region, increasing from northwest to southeast, with approximately 500 mm falling annually, mostly during summer months. The distribution of water resources is uneven, influenced by both geographical features and seasonal rainfall patterns.

The northwest of this region boasts higher terrain, leading to reduced evaporation and precipitation. As a vital player in China’s agricultural landscape, it houses over 7 million hectares dedicated to grain crops, primarily wheat and maize, which are crucial for local food security. Despite its significant contributions to grain production, the BTH region, spanning over 200,000 square kilometers and home to more than 100 million people, faces some of the country’s most pressing environmental challenges [26]. The commitment to high agricultural yields in recent years, reliant on excessive irrigation and fertilizer, has generated numerous regional environmental detriments. The sustainability of agricultural output is further threatened by a disconnect between crop production systems and local environmental conditions, which exacerbates associated risks.

### Multi-source data

The daily climatic variables from 112 meteorological stations in the BTH region of China from 1981 to 2020 were downloaded from the China Meteorological Data Sharing Service System. The climate database consists of local latitude, precipitation, solar radiation, and temperatures (minimum, maximum, and annual average).

Crop observation data (winter wheat and summer maize) during 1981-2020 were gathered from local agricultural meteorological experimental stations. At each experimental station, the technological staffs recorded some important information about agricultural processes, including yield of winter wheat and summer maizes, yield components, and dates of planting, emergence, booting, and maturity, and so on.

The China Soil Science Database (http://www.soil.csdb.cn/) provided most of the soil data. The bulk density of soil (g·cm^-3^), saturated water content (mm·mm^-1^), field capacity (mm·mm^-1^), wilting coefficient (mm·mm^-1^), air dry coefficient (mm·mm^-1^), total nitrogen content (%), pH value, and other variables made up of the soil data. The date, depth, density and spacing of sowing, the date and the amount of fertilizing and irrigating, etc., made up most of the management data. These data were mainly gathered from previous research results and local cultivation experiences.

### Cropping systems

This study adopted three cropping systems, including spring maize (M, one crop a year), winter wheat–summer maize (WM, two crops a year) and winter wheat–summer maize–spring maize (WMM, three crops in two years) (Table 1). According to local planting practices [2], the popular varieties of wheat (Kenong 1993) and maize (Zhengdan 958) were used. Spring maize was sown in April and harvested in October of that year. In October of that year, the winter wheat in this area was planted and harvested in June of the next year. In October of the following year, summer maize was planted and harvested.

**Table 1:** Agronomic management and fertilization treatments under different cropping systems.

Cropping system	Crop (variety)	Sowing date	Planting depth (cm)	Row distance (cm)	Fertilization treatment (kg·ha^-1^)			
					F1	F2	F3	F4
Spring maize (M)	Spring maize (Zhengdan 958)	June 10th	3	60	0	60	120	150
Winter wheat-Summer maize (WM)	Winter wheat (Kenong 1993)	October 15th	5	35	0	90	180	250
	Summer maize (Zhengdan 958)	June 10th	3	60	0	60	120	150
Winter wheat-Summer maize- Spring maize (WMM)	Winter wheat (Kenong 1993)	October 15th	5	35	0	90	180	250
	Summer maize (Zhengdan 958)	June 10th	3	60	0	60	120	150
	Spring maize (Zhengdan 958)	April 25th	3	60	0	60	120	150

### Fertilization treatments

Four fertilization treatments were set based on local planting experience and existing research results [27–29]. F1 treatment was no fertilization. F2 treatment referred to applying 90 kg·ha^-1^ and 60 kg·ha^-1^ base fertilizer before sowing for winter wheat and summer maize, respectively. The F3 treatment included giving 180 kg·ha^-1^ of base fertilizer for winter wheat and 120 kg·ha^-1^ for summer maize ahead of sowing. The F4 treatment consisted of carrying out 250 kg·ha^-1^ of base fertilizer for winter wheat and 150 kg·ha^-1^ for summer maize prior to sowing. The specific fertilization information under different cropping patterns was also shown in Table 1.

### APSIM model

The Agricultural Production Systems sIMulator (APSIM) is the general term for the Australian crop model series, which is utilized by more than 110 nations globally [30] and has been generally tried and used to study the relationships between crop and environment in China [31,32]. Climate, crops, soil water, and soil nitrogen are just a few of its submodules. It has strong dynamic simulation capabilities in crop yield, soil carbon and nitrogen, and GHG emissions. Therefore, APSIM is popularly utilized in crop rotation, water resource balance, environmental changes, and so on [33].

In APSIM-SoilN module, the soil’s organic matters are separated into the fresh organic matter (FOM), biomass (BIOM), and humus (HUM) components. The changes in soil nitrogen and carbon are daily simulated through the association estimations among the three sections. Particularly, the carbon contents of FOM and HUM are the primary determinants of SOC. The changes in ammonium ion concentration, nitrate ion concentration, soil temperature and soil water are primarily responsible for the changes in soil nitrogen content. In the APSIM-SoilN model, nitrogen transformation is divided into nitrification and denitrification processes. Specifically, nitrous oxide (N₂O) emissions from denitrification are mainly simulated and estimated using APSIM model. The simulation also incorporates the denitrification rate and the N₂/N₂O ratio generated during the denitrification process. In addition, the proportion of nitrification nitrogen is then used to calculate the N_2_O produced by nitrification process [34].

### Parameter determination and validation of APSIM model

Several modules of the APSIM model were used in this study, including meteorological module (Met), time module (Clock), soil module (Soil), fertilization module (Fertilize), management module (Manager), and result output module (Outputfile). The parameters of the APSIM model were calibrated using observational data from 2014 to 2015. Then, the APSIM model was validated using observational data from 2016 to 2020 obtained from 9 representative agricultural meteorological observation stations in Gaocheng, Rongcheng, Luanzhou, Miyun, Feixiang, Sanhe, Jixian, Jinghai, and Baodi.

In the APSIM model, there are two primary categories of variety control parameters: one regulates crop development and growth, while the other governs the formation of crop yield. The APSIM model divides the growth and development of wheat and maize into 11 stages and 10 stages, respectively. For wheat, developmental progression at all stages except the sowing–seedling stage (which is controlled by soil moisture) is predominantly governed by vernalization coefficient, photoperiod, and accumulated temperature. By contrast, the developmental stages of maize are mainly determined by accumulated temperature and photoperiod. The yield formation of both crops is simulated using the intrinsic distribution coefficients embedded in the APSIM model.

In this study, all model parameters were determined via a trial-and-error approach, with soil parameters finalized and cultivar control parameters calibrated across the entire growth period from sowing to harvest. In addition, the agreement index (D) was used to evaluate the agreement between observed and simulated values. The formula is as follows:


$$\begin{array}{c} D=\text{1}-\frac{\sum {(Y_{obs}-Y_{sim})}^{\text{2}}}{\sum (\left|Y_{sim}-Y_{mean}\right|+{|Y_{obs}-Y_{mean}|}^{\text{2}})} \end{array}$$ (1)

Where *D* is the agreement index, ranging from 0 (no agreement) to 1 (perfect agreement); *Y*_*obs*_ is the observed value; *Y*_*sim*_ is the simulated value; *Y*_*mean*_ is the arithmetic mean of all observed values.

The model performance was classified into four grades: Excellent (D ≥ 0.90), Good (0.70 ≤ D < 0.90), Moderate (0.50 ≤ D < 0.70), and Poor (D < 0.50).

### Crop climate resilient index

To quantify the climate resilience of agricultural systems and simultaneously consider production functions and environmental effects, a crop climate resilience index was constructed based on four core variables: yield, SOC, SGHG and N₂O, so as to realize the coordinated evaluation of production stability and environmental sustainability.

### Indicator selection and ecological significance

The selected four variables cover the production and environmental ends of the agricultural system, and the ecological significance of each indicator is as follows: ① yield: It characterizes the stable and guaranteed production capacity of the agricultural system under climate disturbances, which is the core embodiment of production resilience. The higher the indicator value, the stronger the production stability; ② SOC: It reflects the soil fertility level, carbon sequestration potential and ecological buffering capacity. As a key indicator of soil ecosystem resilience, the higher its content, the stronger the buffering capacity of the soil against climate fluctuations; ③ Total SGHG emissions: It includes the main greenhouse gases emitted from farmland soils, characterizing the environmental pressure of the agricultural system. The lower the total emissions, the better the environmental resilience; ④ N₂O: It is a potent greenhouse gas in the farmland ecosystem. As the main non-CO₂ greenhouse gas emission source, the lower its emission intensity, the more environmentally friendly the agricultural system.

### Data standardization

Due to the significant differences in the dimensions of the four variables, to eliminate the influence of dimensions and ensure the rationality of index integration, all indicators were normalized to the interval of 0 ~ 1, which were calculated separately as positive indicators and negative indicators:

### Positive indicators

Positive indicators include crop yield and SOC. The standardization formula is as follows:


$$\begin{array}{c} X_{norm}=\frac{X-X_{min}}{X_{max}-X_{min}} \end{array}$$ (2)

Where *X*_*norm*_ is the standardized value of the indicator, the higher the indicator value, the stronger the resilience; *X* is the original value of the indicator; *X*_*max*_ and *X*_*min*_ are the maximum and minimum values of the indicator in the study area, respectively.

### Negative indicators

Negative indicators include total SGHG emissions and N₂O emissions. The standardization formula is as follows:


$$\begin{array}{c} X_{norm}=\text{1}-\frac{X-X_{min}}{X_{max}-X_{min}} \end{array}$$ (3)

The meanings of each parameter in the formula are the same as those in the standardization formula of positive indicators. The lower the indicator value, the stronger the resilience.

### Climate resilience index

The analytic hierarchy process method is used to determine the weight of each indicator. Combined with the principle of production priority and ecological coordination of the agricultural system, and comprehensively considering the ecological and production value of each indicator, the climate resilience index (I) is integrated by the weighted sum method, and the calculation formula is as follows:


$$\begin{array}{c} I=w_{\text{1}}Y_{norm}+w_{\text{2}}SOC_{norm}+w_{\text{3}}SGHG_{norm}+w_{\text{4}}N_{\text{2}}O_{norm} \end{array}$$ (4)

Where *w*_1_, *w*_2_, *w*_3_, and *w*_4_ are the weights of crop yield, SOC, SGHG, and N₂O, respectively; *Y*_*norm*_, *SOC*_*norm*_, *SGHG*_*norm*_, and *N*_*2*_*O*_*norm*_ are the standardized values of each indicator, respectively.

## Results

### Assessing the validity and regional adaptability of APSIM

#### Crop parameters determination.

In this study, a systematic trial-and-error calibration approach was adopted to optimize the cultivar-specific parameters required for the APSIM model. To ensure the model can accurately reproduce local crop growth dynamics and yield performance under regional climatic and soil conditions, key growth and development parameters covering the complete growing season from sowing to harvest were adjusted and finalized for typical crop cultivars widely planted in the study area, including winter wheat (Kenong 1993), summer maize (Zhengdan 958), and early maize (Zhengdan 958). All calibrated cultivar parameters that govern phenological development, biomass accumulation, and yield formation are summarized in detail in Table 2, which provides a fundamental parameter basis for subsequent model validation, simulation analysis, and regional adaptability evaluation of the APSIM model.

**Table 2:** Calibrated cultivar-specific parameters for the APSIM model.

Crop variety	Parameter name	Value	Units
Wheat (Kenong 1993)	Vernalization sensitivity	2.5	/
	Photoperiod sensitivity	2.9	/
	Grain-filling thermal time	585	℃**·**d
	Potential grain-filling rate	0.0025	/
	Grains per gram stem	40	number
Maize (Zhengdan 958)	Thermal time: emergence to juvenile end	282.5	℃·d
	Thermal time: flowering to maturity	800	℃·d
	Thermal time: flowering to grain-filling start	140	℃·d
	Maximum grains per plant	655	number
	Potential grain growth rate	10.75	/
	Photoperiod critical point 1	12.5	hours
	Photoperiod critical point 2	23.5	hours
	Photoperiod response slope	21	/

#### Model validation.

To comprehensively evaluate the predictive performance and applicability of the APSIM model, multi-dimensional validation was conducted based on observed crop phenology and yield data. Based on the APSIM-Wheat and APSIM-Maize modules, the key phenological periods (sowing to flowering and sowing to maturity) and final yields of winter wheat and summer maize at nine agro-meteorological stations across the Beijing-Tianjin-Hebei (BTH) region for the period 2016–2020 were simulated.

The index of agreement (D) was adopted to quantitatively assess the consistency between simulated and measured values for both crops. For winter wheat, the D values were 0.9784 for the flowering stage, 0.9535 for the maturity stage, and 0.7897 for yield. For summer maize, the corresponding D values were 0.9703, 0.9529, and 0.8001, respectively. These results demonstrated excellent performance of the APSIM model in simulating crop phenological processes and satisfactory accuracy in yield prediction, verifying the reliability and applicability of the calibrated model for further scenario simulations and quantitative analysis (Table 3).

**Table 3:** Model performance evaluation using the agreement index.

Crop	Evaluation indicator	Agreement index (D)	Performance rating	Sample number
Wheat	Days from sowing to flowering	0.9784	Excellent	28
	Days from sowing to maturity	0.9535	Excellent	28
	Yield	0.7897	Good	28
Maize	Days from sowing to flowering	0.9703	Excellent	27
	Days from sowing to maturity	0.9529	Excellent	27
	Yield	0.8001	Good	27

### Effect of management measures on soil organic carbon (SOC) under different cropping systems

**Fig 1:**
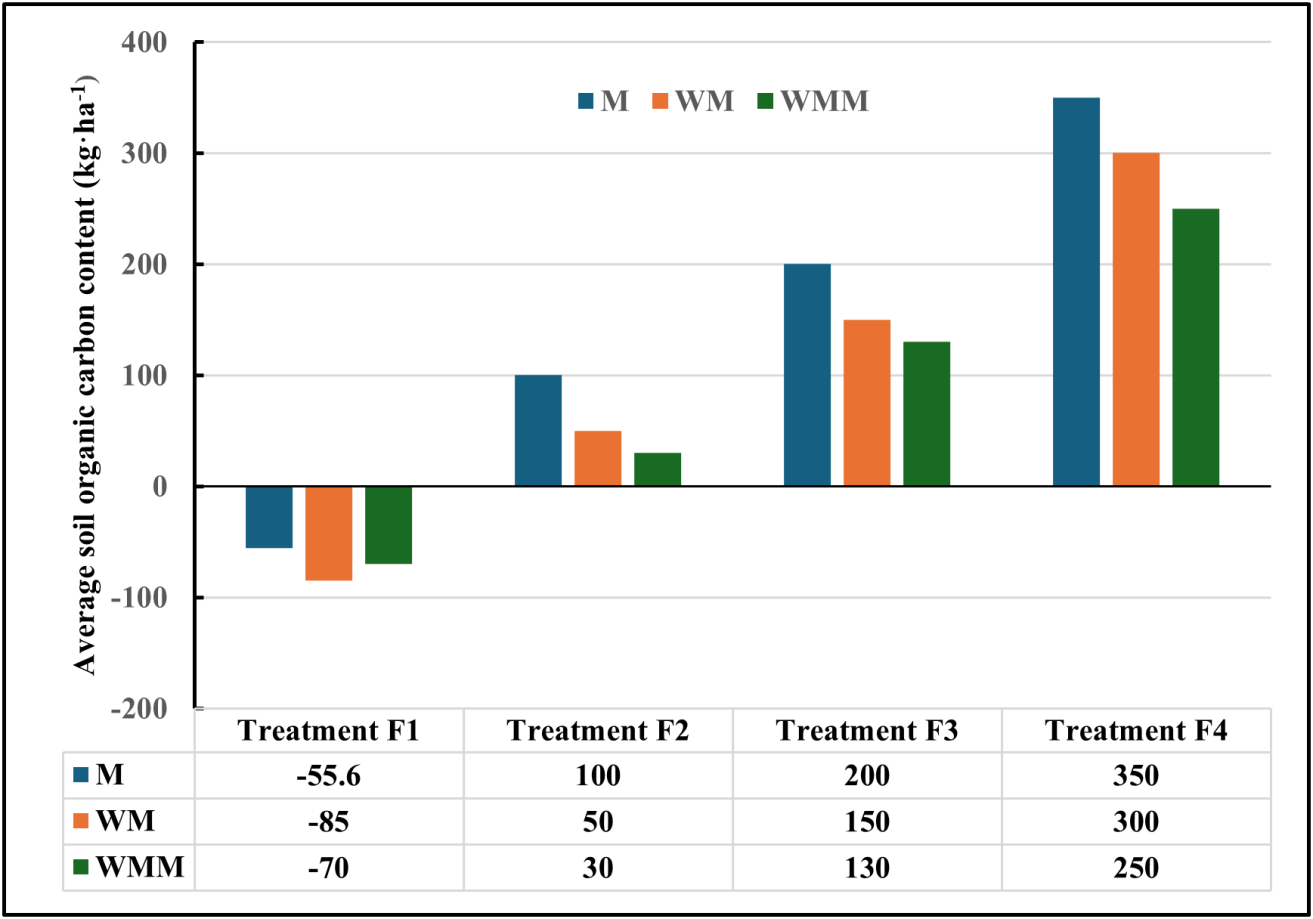
Average soil organic carbon (SOC) content during 1981-2020 in different cropping systems under four fertilization treatment.

Fig 1 compares the effect of four fertilization management treatments on SOC across three cropping systems. The additional SOC results are shown in S1 Data in the Supporting Information. Treatment F1 showed the lowest SOC levels in all systems. Treatments F2 and F3 introduced moderate inputs, leading to noticeable SOC increases, especially in the WM and WMM cropping systems. Treatment F4, with the highest input, consistently produced the highest SOC content across all systems, reaching 350 kg·ha ⁻ ¹ (M), 300 kg·ha ⁻ ¹ (WM), and 250 kg·ha ⁻ ¹ (WMM). This indicated that intensive fertilization practices significantly enhanced soil carbon sequestration, particularly in more diverse cropping systems. Overall, the positive effect of fertilization measures on SOC was consistent but varied in magnitude depending on the cropping system, with more complex rotations (WMM) responding most strongly to high-input treatments.

In addition, the interannual variations in SOC content between 1981 and 2020 in the study area were simulated by APSIM model at three cropping systems and four fertilization treatments. For the M cropping system, the annual variation in SOC content increased with the increase in fertilizer application, and the annual variation trends of the three fertilization treatments (F2, F3, and F4) were similar. The variation in SOC was higher between 1982 and 1996, and then gradually decreased and stabilized. For the WM cropping system, the annual variation trend of SOC content under the four fertilization treatments was consistent, with a relatively stable trend before 2000 and a downward trend after 2000. For the WMM cropping system, the annual variation trend of SOC content was relatively smooth, with a small variation range.

In conclusion, throughout the previous 40 years, the average SOC content across three cropping systems of M, WM, and WMM grew dramatically as fertilizer application increased. The average SOC contents of WM system were the highest, and they were −74.3 kg·ha^-1^, 45.1 kg·ha^-1^, 125.6 kg·ha^-1^, and 187.9 kg·ha^-1^ under F1, F2, F3, and F4 fertilization treatments, respectively. The average SOC contents of M system were the second highest, and they were −91.1 kg·ha^-1^, 122.8 kg·ha^-1^, 258.1 kg·ha^-1^, and 294.9 kg·ha^-1^ under F1, F2, F3, and F4 fertilization treatments, respectively. The annual average SOC contents of WMM system were the lowest, and the annual average SOC contents under F1, F2, F3, and F4 fertilization treatments were −93.1 kg·ha^-1^, 30.6 kg·ha^-1^, 97.5 kg·ha^-1^, and 113.5 kg·ha^-1^, respectively.

In terms of spatial distributions, the overall average SOC contents under different systems between 1981 and 2020 in the BTH region were higher in the North and lower in the South. For the M cropping system, under F1 fertilization treatment, the average SOC contents changing from −102.5 kg·ha^-1^ to 9.4 kg·ha^-1^ were lower in the southern Shijiazhuang, and higher in the central and northern BTH region. Under F2, F3, and F4 fertilization treatments, the average SOC contents were higher in the northern BTH region, and were lower in the central and southern BTH region. In particular, the low value areas were all located in the southern region of Shijiazhuang, and the average SOC contents under F2, F3, and F4 treatments were −254.1 kg·ha^-1^ ~ 511.2 kg·ha^-1^, −285.3 kg·ha^-1^ ~ 680.1 kg·ha^-1^, and −158.1 kg·ha^-1^ ~ 688.6 kg·ha^-1^, respectively.

For the WM cropping system, under four fertilization treatments, the central and eastern regions of Shijiazhuang had the lowest average SOC content, while the highest SOC content was found in northern part of Beijing, followed by the southern BTH region. Specifically, the average SOC contents under the F1, F2, F3, and F4 treatments were −193.2 kg·ha^-1^ ~ 72.4 kg·ha^-1^, −145.6 kg·ha^-1^ ~ 493.2 kg·ha^-1^, −93.5 kg·ha^-1^ ~ 750.4 kg·ha^-1^ and −40.5 kg·ha^-1^ ~ 809.5 kg·ha^-1^.

For the WMS cropping system, under F1 fertilization treatment, the average SOC contents changed from −143.2 kg·ha^-1^ to 12.6 kg·ha^-1^, with lower SOC in the southern Shijiazhuang and the central BTH region and higher SOC in the northern BTH region. Under F2 fertilization treatment, the average SOC content varying between −67.4 kg·ha^-1^ ~ 195.5 kg·ha^-1^ were higher in the northern BTH region and lower in the central and southern BTH region. Under F3 and F4 fertilization treatments, the average annual SOC contents were −10.3 kg·ha^-1^ ~ 270.4 kg·ha^-1^ and 3.4 kg·ha^-1^ ~ 291.6 kg·ha^-1^, respectively.

### Effect of management measures on nitrous oxide (N_2_O) under different cropping systems

**Fig 2:**
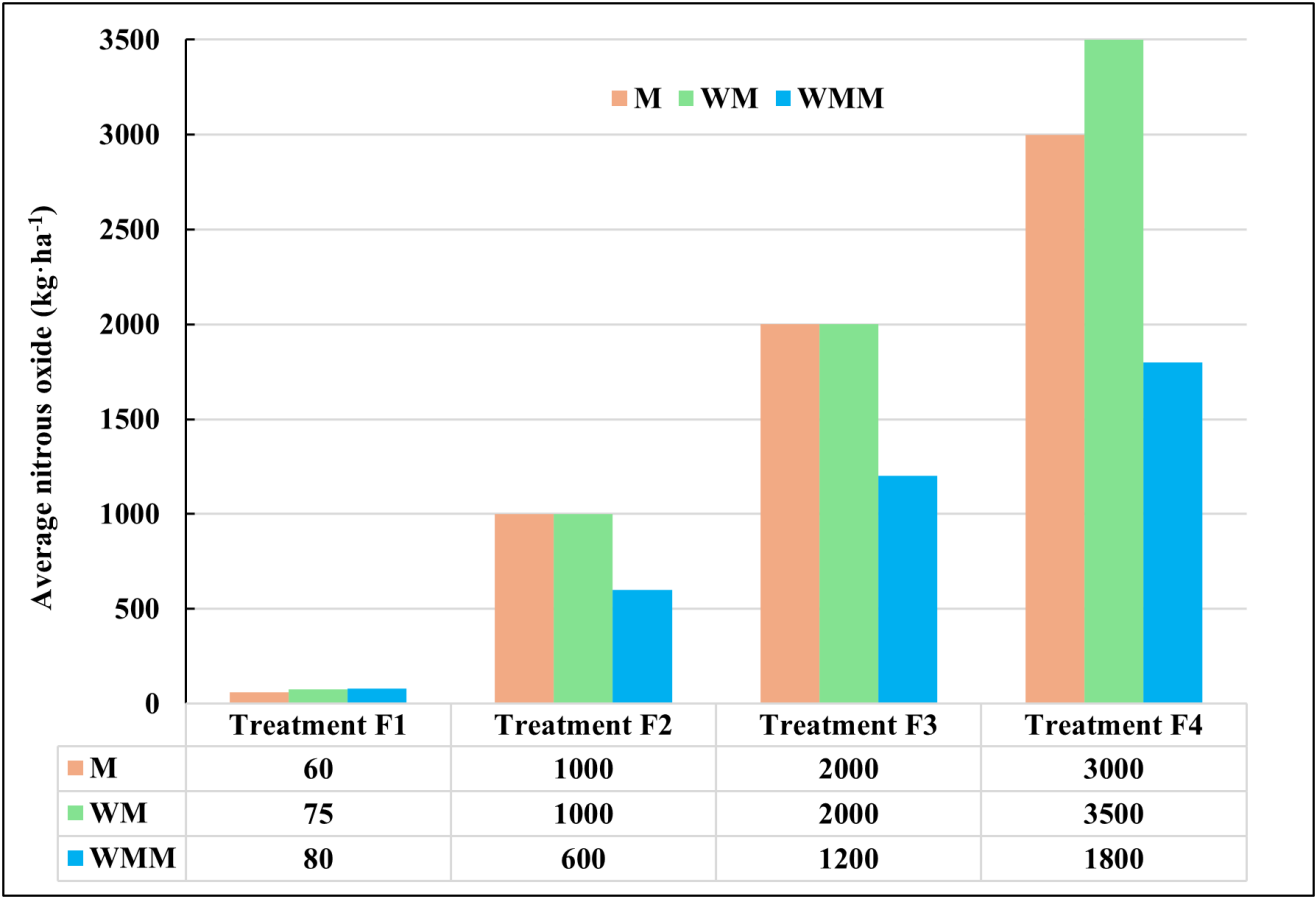
Average nitrous oxide (N_2_O) during 1981-2020 in different cropping systems under four fertilization treatment.

In the BTH region, the average N_2_O emissions from three cropping systems steadily increased over the last 40 years under four different fertilization treatments (Fig 2).Additional N_2_O emissions results are presented in S2 Data (Supporting Information). F1 fertilization treatment showed relatively low N₂O levels across all systems, with an average ranging from 10 kg·ha ⁻ ¹ to 20 kg·ha ⁻ ¹. Under F1 fertilization treatment, the N_2_O emissions from three cropping systems during 1981-2020 were 17.4 kg·ha^-1^ ~ 154.9 kg·ha^-1^, 24.1 kg·ha^-1^ ~ 107.7 kg·ha^-1^ and 40.1 kg·ha^-1^ ~ 138.9 kg·ha^-1^, correspondingly. Treatments F2 and F3 led to substantial increases in N₂O emissions, particularly under WM and WMM systems, with F3 reaching 2000 kg·ha ⁻ ¹ under both WM and WMM. Treatment F4, with the highest input, produced the highest N₂O emissions in all cropping systems, peaking at 3500 kg·ha ⁻ ¹ under WM and 1800 kg·ha ⁻ ¹ under W cropping systems. Especially, the N_2_O emissions from three cropping systems between 1981–2020 under F4 fertilization treatment were 636.4 kg·ha^-1^ ~ 2332.3 kg·ha^-1^, 1812.9 kg·ha^-1^ ~ 6850.3 kg·ha^-1^ and 1197.9 kg·ha^-1^ ~ 3055.1 kg·ha^-1^, respectively. Overall, increased fertilization management intensity consistently raised N₂O emissions, with the WM system being the most responsive. This suggests a trade-off between carbon sequestration and greenhouse gas mitigation.

The spatial distribution of N₂O emissions exhibited substantial variations across the study area. For the M cropping system, the BTH’s center and eastern regions had lower N_2_O emissions than its southern and northern regions. The average N2O emissions throughout the last four decades under F1 fertilization treatment were lower in the eastern and northwest regions of BTH and greater in the southern BTH, ranging from 25.9 kg·ha^-1^ to 93.3 kg·ha^-1^. Under F2 fertilization treatment, the average N_2_O emissions ranging from 177.2 kg·ha^-1^ to 1069.3 kg·ha^-1^ were higher in the northern and northwestern regions of BTH, followed by the central and eastern part of BTH. Under both F3 and F4 treatments, N₂O emissions decreased in the central and eastern areas of the BTH region, whereas higher N₂O emissions were observed in the northern and southern regions. The average N_2_O emissions during 1981-2020 under the F3 and F4 treatments were 431.0 kg·ha^-1^ ~ 4181.5 kg·ha^-1^ and 590.0 kg·ha^-1^ ~ 5915.2 kg·ha^-1^, respectively.

As for the WM cropping system, under the F1 treatment, high average N_2_O emissions were concentrated in the southern BTH region and eastern Shijiazhuang, while the remaining areas exhibited lower emissions, ranging from 33.5 kg·ha^-1^ to 85.6 kg·ha^-1^. From 1981 to 2020, the average N_2_O emissions under the F2 treatment varied between 522.9 kg·ha^-1^ and 4098.5 kg·ha^-1^, with comparatively uniform ranges. Under the F3 treatment, the average N_2_O emissions were higher in the northern part, and lower in the eastern BTH and some parts of Tianjin, ranging from 1962.3 kg·ha^-1^ to 4461.6 kg·ha^-1^. Under the F4 treatment, the average N_2_O emissions were higher in the northern, northwest and southern parts of BTH, and lower in the eastern part of BTH, changing from 3251.2 kg·ha^-1^ to 6497.7 kg·ha^-1^.

As for the WMM cropping system, under the F1 fertilization treatment, the northern BTH region exhibited the highest annual average N_2_O emissions, followed by the southeN2rn BTH region and eastern Shijiazhuang. In contrast, eastern BTH presented relatively low annual N_2_O emissions, ranging from 55.5 kg·ha^-1^ to 186.4 kg·ha^-1^. Under the F2, F3, and F4 fertilization treatments, the higher annual average N_2_O emissions were mainly distributed in the northern, southern, and northwestern BTH regions, with emission ranges of 246.8 kg·ha^-1^ ~ 630.2 kg·ha^-1^, 511.6 kg·ha^-1^ ~ 2130.1 kg·ha^-1^, and 902.3 kg·ha^-1^ ~ 3009.8 kg·ha^-1^, respectively.

### Effect of management measures on soil greenhouse gas (SGHG) emissions under different cropping systems

**Fig 3:**
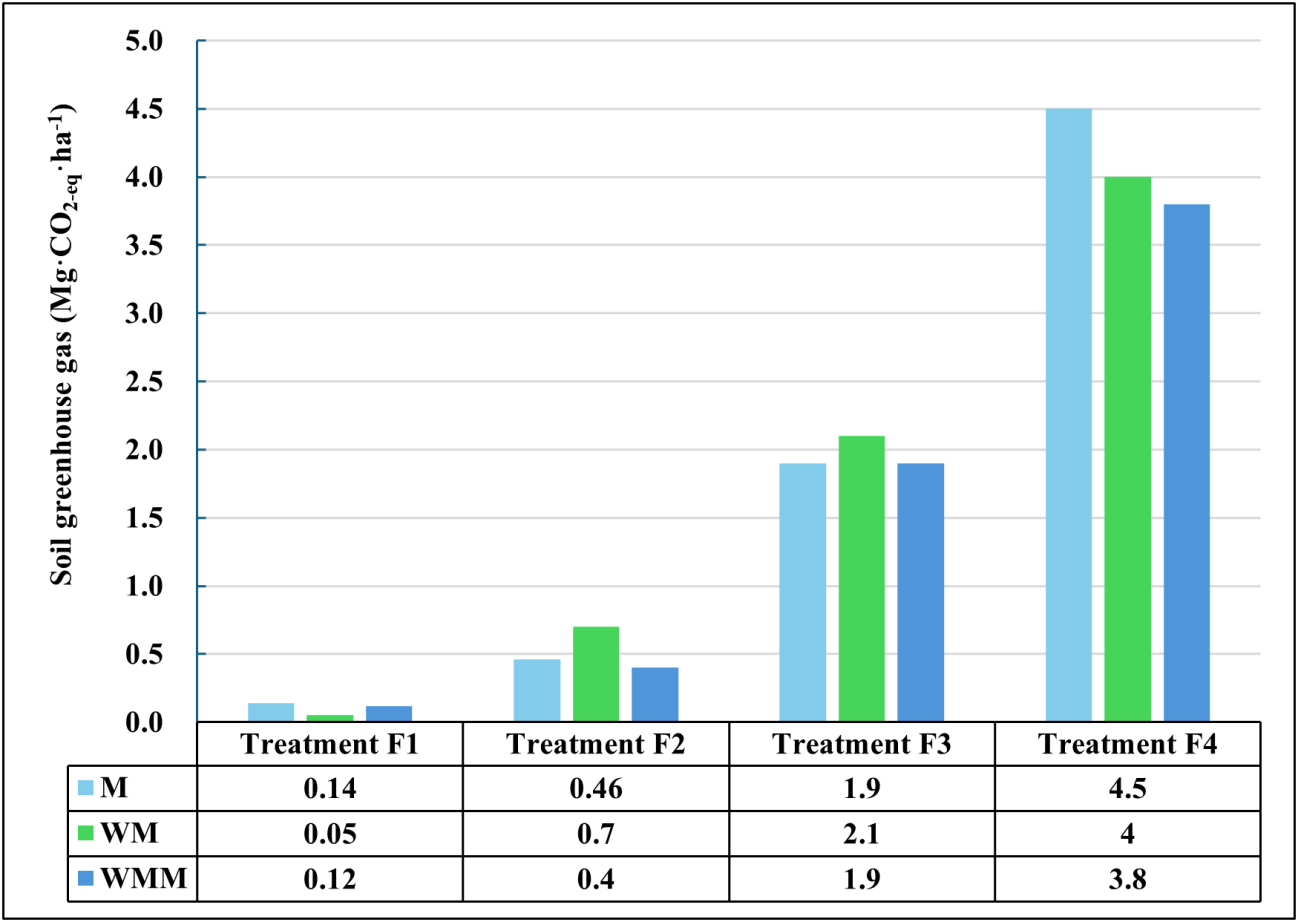
Average soil greenhouse gas (SGHG) emissions during 1981-2020 in different cropping systems under four fertilization treatment.

Under different fertilization measures, the SGHG emissions from three cropping systems showed fluctuating and increasing trend from 1981 to 2020. With the increase of fertilization amount, the interannual variations of SGHG emissions significantly increased from 0.2 Mg·CO_2-eq_·ha^-1^ ~ 0.8 Mg·CO_2-eq_·ha^-1^ under F1 fertilization treatment to 2.2 Mg·CO_2-eq_·ha^-1^ ~ 10.2 Mg·CO_2-eq_·ha^-1^ under F4 fertilization treatment (Fig 3). Additional SGHG emissions results are presented in S3 Data (Supporting Information).

For the M cropping system, the SGHG emissions under F2, F3, and F4 fertilization treatments were lower than those under F1 fertilization treatment before 1998. After that, the SGHG emissions gradually increased over time. Particularly, from 1981 to 2020, the SGHG emissions under the F2 fertilization treatment exhibited a modest upward trend, whereas the SGHG emissions under the F3 and F4 fertilization treatments clearly indicated upward trends. Under the F1 treatment, the SGHG emission was 0.3 Mg·CO_2-eq_·ha^-1^ on the average, with a range of 0.3 to 0.5 Mg·CO_2-eq_·ha^-1^. Under the F2 treatment, the ranges of SGHG emission were −0.2 Mg·CO_2-eq_·ha^-1^ to 1.4 Mg·CO_2-eq_·ha^-1^, exhibiting an average of 0.3 Mg·CO_2-eq_·ha^-1^. Under the F3 treatment, the SGHG emission fluctuated from −0.4 Mg·CO_2-eq_·ha^-1^ to 3.1 Mg·CO_2-eq_·ha^-1^, and averagely was 0.7 Mg·CO_2-eq_·ha^-1^. Under the F4 treatment, the SGHG emission was 1.1 Mg·CO_2-eq_·ha^-1^, and extended between −0.2 Mg·CO_2-eq_·ha^-1^ and 3.9 Mg·CO_2-eq_·ha^-1^.

For the WM cropping system, under F1 and F2 treatments, the SGHG emissions during 1981-2020 fluctuated less over time. Nevertheless, under F3 and F4 treatments, the SGHG emissions showed significant increasing trends with large interannual variations. Especially, under F1 treatment, the interannual variation of SGHG emission was small, ranging from 0.2 Mg·CO_2-eq_·ha^-1^ to 0.8 Mg·CO_2-eq_·ha^-1^, with an average of 0.4 Mg·CO_2-eq_·ha^-1^. Under the F2 treatment, the range of SGHG emission varied from 0.1 Mg·CO_2-eq_·ha^-1^ to 2.6 Mg·CO_2-eq_·ha^-1^, with an average of 0.8 Mg·CO_2-eq_·ha^-1^. Under the F3 treatment, the interannual variations of SGHG emission were 1.0 Mg·CO_2-eq_·ha^-1^ to 6.8 Mg·CO_2-eq_·ha^-1^, with an average of 3.1 Mg·CO_2-eq_·ha^-1^. The SGHG emission during F4 treatment was 5.8 Mg·CO_2-eq_·ha^-1^, fluctuating between 2.2 Mg·CO_2-eq_·ha^-1^ and 10.2 Mg·CO_2-eq_·ha^-1^,

For the WMM cropping system, the trends of interannual variation in SGHG emissions were comparable to those in the WM cropping system. While the growing trends of SGHG emissions over time were evident under F3 and F4 treatments, the interannual changes of SGHG emissions under F1 and F2 treatments were comparatively mild. The interannual variation ranges of SGHG emissions under F1, F2, F3, and F4 treatments were 0.3 Mg·CO_2-eq_·ha^-1^ ~ 0.6 Mg·CO_2-eq_·ha^-1^, 0.2 Mg·CO_2-eq_·ha^-1^ ~ 1.6 Mg·CO_2-eq_·ha^-1^, 0.6 Mg·CO_2-eq_·ha^-1^ ~ 3.6 Mg·CO_2-eq_·ha^-1^, and 1.3 Mg·CO_2-eq_·ha^-1^ ~ 5.1 Mg·CO_2-eq_·ha^-1^, with average values of 0.4 Mg·CO_2-eq_·ha^-1^, 0.6 Mg·CO_2-eq_·ha^-1^, 1.7 Mg·CO_2-eq_·ha^-1^, and 3.1 Mg·CO_2-eq_·ha^-1^, respectively.

The annual average SGHG emissions and their variation coefficient under different fertilization measures. The average SGHG emissions increased with the increase of fertilizer application, and the variation coefficients firstly increases and then decreased with the increase of fertilizer application. From 1981 to 2020, among the four fertilization treatments, the F4 fertilization treatment had the highest average SGHG emissions. The average SGHG emissions of the M, WM, and WMM cropping systems were 1.0 Mg·CO_2-eq_·ha^-1^, 5.8 Mg·CO_2-eq_·ha^-1^, and 3.1 Mg·CO_2-eq_·ha^-1^, respectively. The average SGHG emissions of the M, WM, and WMM cropping systems under the F3 fertilization treatment were 0.7 Mg·CO_2-eq_·ha^-1^, 3.2 Mg·CO_2-eq_·ha^-1^, and 1.7 Mg·CO_2-eq_·ha^-1^, respectively. The SGHG emissions of M, WM, and WMM cropping systems under F2 fertilization treatment were 0.3 Mg·CO_2-eq_·ha^-1^, 0.8 Mg·CO_2-eq_·ha^-1^, and 0.6 Mg·CO_2-eq_·ha^-1^, respectively. However, the average SGHG emissions of the M, WM, and WMM cropping systems under F1 fertilization treatment were the lowest, with average values of 0.3 Mg·CO_2-eq_·ha^-1^, 0.4 Mg·CO_2-eq_·ha^-1^, and 0.4 Mg·CO_2-eq_·ha^-1^, respectively.

### Effect of management measures on crop total yield under different cropping systems

**Fig 4:**
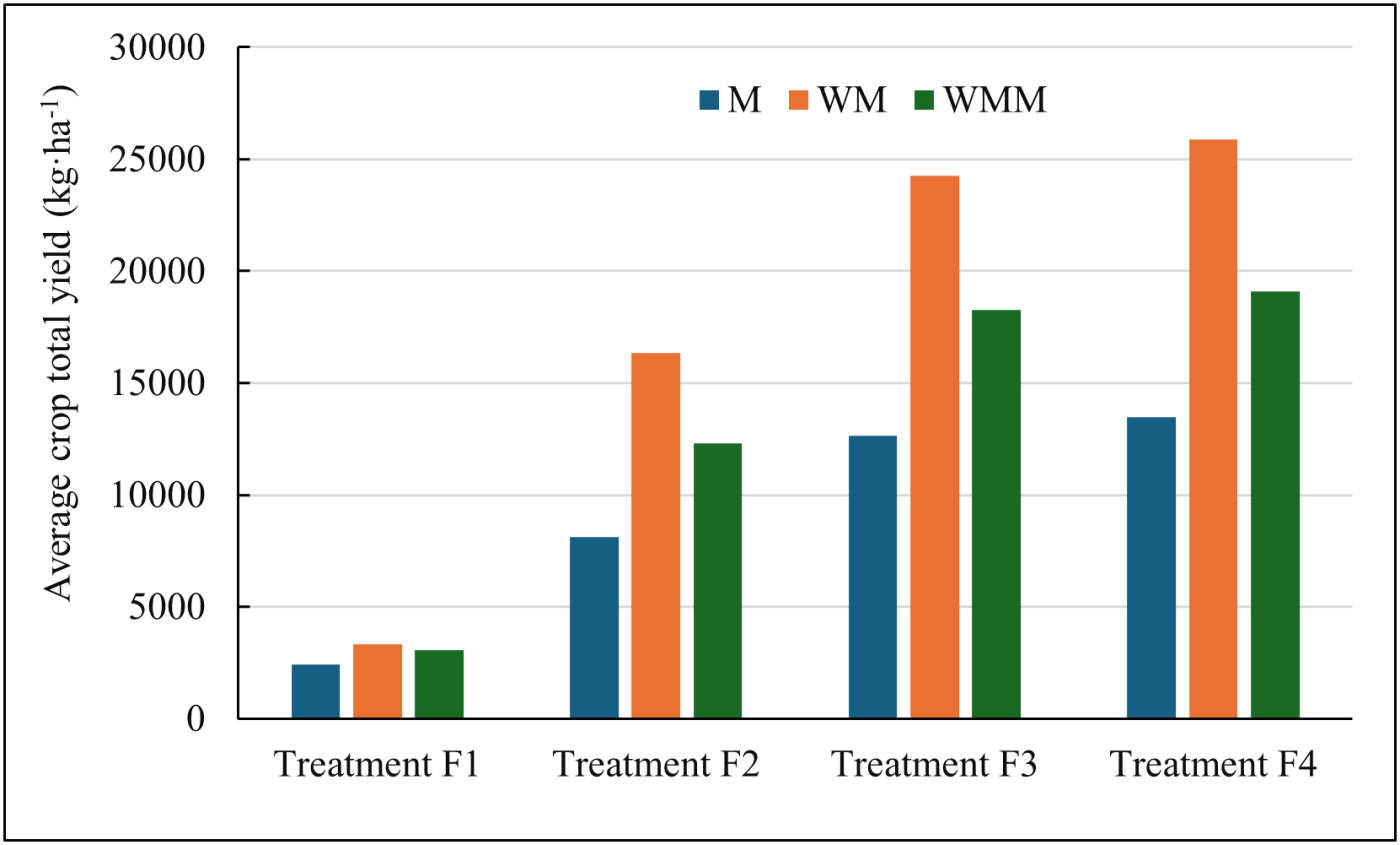
Average crop total yield during 1981-2020 in different cropping systems under four fertilization treatment.

Fig 4 presents average crop total yields across three cropping systems and four fertilizer treatments during 1981-2020 in the BTH region. Key trends showed that the average crop total yield increased consistently with fertilizer intensity for all systems, indicating fertilization strongly boosted crop productivity. Among three cropping systems, the WM cropping system consistently outperformed others, with its highest yield (2.59 × 10^4^ kg·ha^-1^ under F4) nearly doubling maximum of M cropping system (1.35 × 10^4^ kg·ha^-1^). The WMM cropping system ranked second, showing intercropping advantages over monoculture. The lowest yields occurred under F1 across all systems, highlighting baseline productivity limitations without adequate fertilization. These results demonstrated that combining appropriate cropping systems with fertilization optimized total crop yield.

### Enhancing climate resilience through optimized cropping systems and nitrogen management

**Fig 5:**
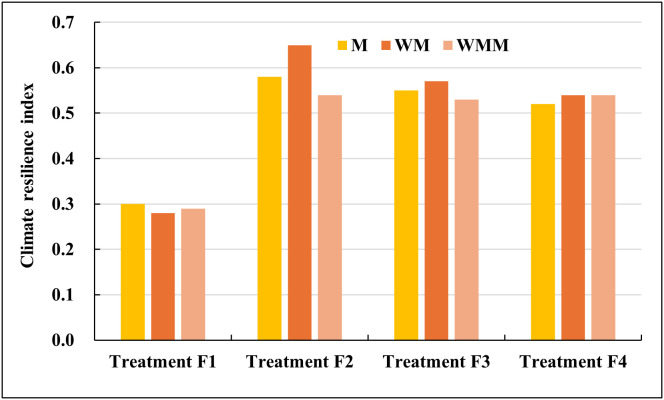
Average climate resilience index during 1981-2020 in different cropping systems under four fertilization treatment.

In this study, the climate resilience index was created to comprehensively evaluate the combined performance of yield, SOC, SGHG, and N₂O across different cropping systems and fertilization treatments during 1981-2020 in the BTH region. Fig 5. shows the climate resilience index values for three cropping systems under four treatments. Additional climate resilience index results are presented in S4 Data (Supporting Information). Under the F1 treatment, all cropping systems exhibited low climate resilience values ranging from 0.28 to 0.30. Specifically, the WM system had the lowest resilience (0.28), followed by WMM (0.29) and M (0.30). This result demonstrates that F1 was the least efficient practice for improving crop climate resilience. The F2 treatment achieved the highest resilience levels, with values reaching 0.58 for the M system and 0.65 for the WM system, while that of the WMM system increased to 0.54. The pronounced resilience elevation from F1 to F2 confirmed that F2 substantially strengthened system resilience, with WM becoming the most resilient cropping system under F2 conditions.

By contrast, F3 and F4 yielded moderate and comparable resilience values (0.52 and 0.57) across all cropping systems. For the M system, resilience under F3 (0.55) and F4 (0.52) was marginally lower than that under F2. A gradual decreasing trend was observed in WM resilience, declining from 0.65 under F2 to 0.57 under F3 and further to 0.54 under F4. In comparison, WMM maintained relatively stable resilience at 0.53 ~ 0.54 across F2 to F4, reflecting steady yet suboptimal performance.

In summary, F2 was identified as the optimal treatment to enhance climate resilience, especially for the WM cropping system. Conversely, F1 fails to effectively improve resilience in all tested systems. Cropping system type exerted distinct effects only under F2, where WM showed superior performance relative to the other two systems. In contrast, all systems presented similar resilience responses under F3 and F4. These conclusions provided practical references for formulating climate-adaptive agricultural management strategies.

## Discussion

### Importance of management practices in agricultural systems

Cropping system adjustments and fertilization measures are essential management strategies for crop growth. Adjusting the cropping system improves land use efficiency, balance soil nutrients, reduce pests and diseases, promote ecological balance, and optimize resource allocation [35]. Fertilization increases crop production by supplementing nutrients that control the crops’ physiological process [36]. This is because nitrogen is a key nutrient element that affects crop growth, development, and organ building. With a certain amount of fertilizer application, it can enhance soil fertility, promote the absorption and accumulation of nitrogen by crop plants, improve the transport of nitrogen from before flowering to grains, and significantly increase yield [37].

Notably, fertilizer is one of the main contributors to SGHG emissions [38]. The amount of nitrate in the soil rises following the addition of nitrogen fertilizer. As the main substrate for soil nitrification and denitrification, the rapid increase of nitrate will inevitably lead to the intensification of nitrification and denitrification processes, thereby increasing the amount of N_2_O produced [39]. In addition, only a portion of the fertilizers that are applied to farmland are absorbed by the crops, the remainder is used by the soil microbes to produce N_2_O. Leaching and volatilization cause some unused nitrogen to be lost, polluting the environments [39].

However, effective fertilization management measures and suitable cropping systems can reduce N_2_O emissions while ensuring high crop yields [26,34–37,40,41]. For example, a study based on field experiments found that 25% reduction in traditional urea combined with organic fertilizers significantly reduced N_2_O emissions and increased by 5.4% of winter wheat yield [42]. Employing mineral nitrogen fertilizer management decreased overall GHG emissions by 6.22% without affecting the rice yield [43]. Intercropping maize and cowpeas, particularly at the row ratio of 1:1, greatly raised the total crop yield by 13.6% in India [44]. Using rotary tillage combined with fertilization and straw return boosted rice and wheat yields by 51% to 69% when compared to rotary tillage alone [45]. However, literature suggested that adding straw to soil increased microbial absorption of mineral nitrogen, reduced the nitrogen available amount for the development and growth of wheat tiller, and thus lowered the effective numbers of spikes and yields [26]. Previous DNDC model simulations of the winter wheat–summer maize rotation system in Hebei Province, China, revealed that optimized fertilization strategies reduced cumulative soil N₂O emissions relative to conventional fertilization, without inducing significant variations in grain yield [46].

The present study focused on two crucial practices that directly influenced crop performances: cropping systems and nitrogen management. Our results indicated that the MS cropping system had the lowest N_2_O emission (57.4 kg·ha^-1^) and SGHG emission (0.3 Mg·CO_2-eq_·ha^-1^) and the highest SOC content (74.3 kg·ha^-1^) when compared to the W and WMM cropping systems. Although the crop yield was slightly lower, it was still ensure stable production. We also highlighted that appropriate fertilization management was necessary to ensure the balance between crop yield and GHG emissions. The highest crop total yield and average SOC content occurred with the F4 fertilization treatment, but there were also increased SGHG and N_2_O emissions. Our research results aligned with previous findings derived from field fixed-point observation experiment and model simulations [26,37,42–46]. Future studies should quantify the respective contributions of nitrification and denitrification to total N_2_O using multi‑model ensemble outputs, and examine how their ratio varies interannually and spatially in response to soil moisture, temperature, and carbon dynamics. This will strengthen mechanistic insights.

### Effects of management practices on SOC and crop yield

As a crucial metric for evaluating the quality and fertility of soil, the SOC content is also a significant way to improve crop yield. The management measures such as appropriate fertilization, planting green manure, and crop rotation, and so on, not only promoted the SOC cycling, but also improved soil fertility, water retention capacity and SOC content. These practices can improve the soil environments and increase the supply of nutrients for crop growth and development, thereby increasing crop yields.

Numerous studies revealed that the crop yields increased with the use of organic and mineral fertilizers, mostly due to enhanced SOC sequestration [47–49]. Our results indicated that average SOC content and crop yield in the BTH region were the highest when treated with F4 fertilization treatment. One plausible explanation was that nitrogen fertilization increased carbon inputs into agroecosystems, providing an effective energy source for soil fauna and thereby promoting SOC accumulation. The improved soil carbon status further enhanced both above‑ground and below‑ground crop biomass, ultimately increasing total crop yield. These findings are supported by the existing results [21,48–51]. For example, a previous study demonstrated that following biochar amendment, the SOC content rose by 26.9% ~ 45.8%, which directly enhanced yield [50]. The soil organic matter increased by 9.33% ~ 11.98% in a three-year organic fertilizer trial on rice fields, significantly increasing the rice yield [21]. Applying 300 kgN·ha^-1^ of nitrogen fertilizer increased the amount of carbon and nitrogen in the soil and plants, as well as the efficiency of nitrogen usage and maize yield, when compared to no fertilizing [51].

### Comprehensive strategies for climate adaptation and implications

Developing cropping systems that are resilient, adaptive, and ecologically sustainable requires integrating tailored agricultural management practices into broader climate change adaptation programs [52–53]. This study identified optimal cropping systems and nitrogen management strategies that enhanced climate resilience by increasing crop yield and reducing SGHG emission. Our findings showed that the F2 fertilization treatment performed optimally across all three cropping system tested. Moreover, the WM cropping system under F2 treatment achieved the highest crop climate resilient index, followed by WMM and M cropping system. Overall, the WM cropping system combined with F2 nitrogen management proved most effective for sustaining crop productivity and reducing N₂O and overall GHG emissions in the BTH region, demonstrating strong potential for climate-smart agriculture.

Previous studies also confirmed that adopting reasonable cropping system practices, which were combined nitrogen fertilizers, increased total crop yield [44,54]. By reducing environmental effects and increasing resource usage efficiency and yield, these practices promoted the sustainable development of agriculture. Additionally, other management strategies such as the 4R nutrient stewardship principle, which refers to the right fertilizer rate, right fertilizer source, right placement, and right timing, could be adopted to optimize nitrogen fertilizer application [55]. It was worth noting that some agronomic improvement measures from different climate-soil-plant-agricultural systems existed spatial adaptability, and should be implemented in accordance with local conditions to maximize efficacy and support climate adaptation [56].

In the upcoming decades, new ways to accomplish the mutually beneficial objectives of environmental sustainability and food security need to be developed and explored, such as enhancements of novel genetic techniques for climate-adapted crop breeding or engineering [57], new microbes with the capability of reducing N_2_O [58], applying coconut husks increasing the consumption of fungal N_2_O producers [59]. Before widespread implementation, the effectiveness and impacts of these novel techniques in diverse environmental settings are suggested to be thoroughly investigated and demonstrated.

## Conclusions

This study investigated climate-smart cropping systems and nitrogen management strategies in China aimed at sustaining crop production while reducing SGHG emissions. The results demonstrated that climate-adaptive cropping systems combined with optimized fertilization were helpful in raising yield stability and mitigate environmental impacts under long-term climate variability. In the BTH region, the SOC content showed a gradual increase from 1981 to 2020 across three cropping systems with elevated fertilizer application. The highest mean SOC content (294.9 kg·ha ⁻ ¹) was observed under the F4 treatment. The SOC distribution was spatially heterogeneous, with higher levels observed in northern areas. Conversely, the N₂O emissions consistently increased over the 40-year period under all fertilization treatments, exhibiting a strong positive correlation with fertilizer input and higher cumulative emissions in the southern part of the region. Crop total yields of wheat and maize responded positively to increased fertilization across all cropping systems. The WM cropping system under the F2 fertilization regime (90 kg·ha ⁻ ¹ for wheat; 60 kg·ha ⁻ ¹ for maize) was identified as the most climate-smart practice. This regime yielded the highest climate resilience index of 0.65, effectively balancing high crop productivity with reduced N₂O and overall SGHG emissions.

In conclusion, this study provides valuable insights into climate-adaptive agricultural practices that can help buffer against the impacts of climate change on regional crop production and contribute to global warming mitigation. Future research should focus on the responsiveness of different crop varieties to N₂O and SGHG emissions, and further explore the integrated effects of factors such as pesticide use, fertilizer application, pest and disease dynamics, production costs, and economic benefits within the water–food–environment nexus.

## Supporting information

S1 Data The data used for average soil organic carbon (SOC) content during 1981-2020 in different cropping systems under four fertilization treatment. (XLSX)

S2 Data The data used average nitrous oxide (N_2_O) during 1981-2020 in different cropping systems under four fertilization treatment. (XLSX)

S3 Data The data used average soil greenhouse gas (SGHG) emissions during 1981-2020 in different cropping systems under four fertilization treatment. (XLSX)

S4 Data The data used average climate resilience index during 1981-2020 in different cropping systems under four fertilization treatment. (XLSX)
